# Wolves on the phone: Public calls reveal a rise in urban concerns as wolves recolonize human-dominated areas

**DOI:** 10.1007/s13280-025-02264-z

**Published:** 2025-10-18

**Authors:** Rudy Brogi, Giovanna Neirotti, Jacopo Cerri, Martina Lazzaroni, Sarah Marshall-Pescini, Luca Mattioli, Marco Apollonio

**Affiliations:** 1 Department of Veterinary Medicine, University of Sassari, via Vienna 2, 07100 Sassari, Italy; 2 NBFC, National Biodiversity Future Center, Piazza Marina 61, 90133 Palermo, Italy; 3 Department of Chemistry, Life Science and Environmental Sustainability, University of Parma, viale Delle Scienze 17/A, 43124 Parma, Italy; 4 Domestication Lab, Konrad Lorenz Institute of Ethology, University of Veterinary Medicine Vienna, Veterinärplatz 1, 1210 Vienna, Austria; 5 Settore Attività Faunistico Venatoria, Pesca Dilettantistica, Pesca in Mare, Regione Toscana, via Arrigo Testa 2, 52100 Arezzo, Italy; 6 Mammal Research Institute, Polish Academy of Sciences, Stoczek 1, 17-230 Białowieża, Poland

**Keywords:** Carnivore, Conservation, Fear, Human dimension, Human–wildlife conflict, Management

## Abstract

European wolf populations are expanding into human-dominated landscapes, triggering novel interactions with citizens and public concerns that may disrupt the traditional urban–rural divide in wolf attitudes and reshape conservation paradigms. We modelled the spatiotemporal distribution and valence of the wolf reports received through a dedicated phone service in Tuscany, Italy (2021–2024). Reports were significantly more common in: (i) late winter, aligning with the peak dispersal period and increased wolf movements. (ii) recently recolonized areas, suggesting a wolf-novelty effect, and (iii) urban areas, where negative valence was also more likely. Public concerns about wolves are increasingly emerging in urban areas, potentially disrupting the traditionally more supportive urban stance on wolf presence. In one of the European regions where wolf recovery began earlier and progressed further, our findings signal a broader shift in public attitudes that may weaken support for wolf conservation, potentially anticipating similar developments in areas of more recent recovery.

**Graphical Abstract:**

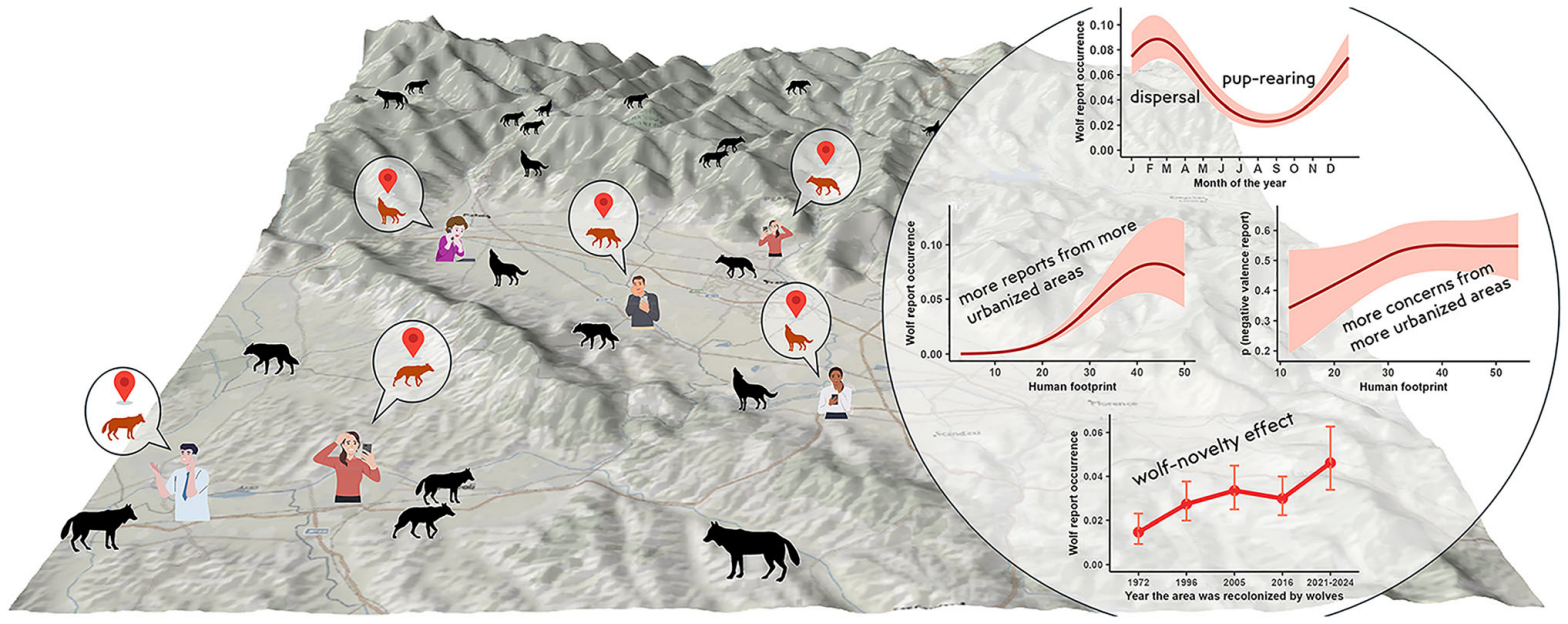

**Supplementary Information:**

The online version contains supplementary material available at 10.1007/s13280-025-02264-z.

## Introduction

European populations of grey wolf (*Canis lupus*) have undergone a remarkable and rapid recovery after a prolonged period of severe persecution that drove them to extinction across much of their historical range (Mech 2017). This recovery, underpinned by legal protection, socio-economic shifts from agriculture and rural practices towards industry, and the rewilding of abandoned rural areas, is celebrated as a conservation success story (Di Bernardi et al. 2025). However, the resurgence of wolf populations also presents significant conservation challenges, particularly in managing conflicts with humans (Mech 2017; Kuijper et al. 2019; Pettersson et al. 2023). The wolves’ return was initially localized in rural areas, where it reignited traditional conflicts with human activities such as livestock breeding and hunting (Ciucci e Boitani 1998; Bisi et al. 2010). The negative impacts of large carnivores being disproportionately experienced in rural communities fostered divergent human–wolf worldviews. This divide often aligns with cultural and political perspectives, putting wolf-opposers who rely on traditional and/or productive rural practices against wolf-protectionists with predominantly urban lifestyles (Ericsson et al. 2018; Pettersson et al. 2021; Barmoen et al. 2024). Over time, however, the wolves’ exceptional adaptability has enabled them to expand beyond natural areas considered their traditional habitat into areas with varying degrees of human dominance (Muhly et al. 2019; Kuijper et al. 2024) characterized not only by productive activities, but also by dense human populations, leading to increasing overlap with both small and large human settlements (Mohammadi et al. 2019; Zanni et al. 2023). As wolves increasingly inhabit areas with higher human densities, urban citizens may begin to directly experience the challenges associated with their presence. Given that positive attitudes towards wolves are linked to greater spatial distance from wolf territories (Zimmermann et al. 2001; Karlsson e Sjöström 2007), this growing proximity has the potential to disrupt the polarized status quo of political views on wolves, possibly bridging the rural–urban divide. By creating a new landscape of concern among citizens towards wolves, this phenomenon highlights a critical yet underexplored aspect of wolf recovery in human-dominated landscapes with the potential to challenge current paradigms of large carnivore management and conservation. Human perceptions of wolves indeed shape political decision-making on conservation measures (Birkland 1998), inform human behaviour towards wolves (Treves et al. 2013), and indicate whether people will support or oppose wolf presence and management strategies (Slagle et al. 2022; Capitain et al. 2025), ultimately shaping the future trajectory of wolf conservation. It is therefore essential to map the spatiotemporal dynamics of these emerging interactions alongside associated public concerns in order to inform effective impact mitigation and conflict minimisation (Kretser et al. 2009; Quinn et al. 2016).

Attitudes towards wildlife are commonly assessed through psychometric questionnaires administered to representative samples of the human population (Whitehouse‐Tedd et al. 2021). While remaining the gold standard for gauging public perceptions, these methods face challenges such as declining response rates and high costs (Beullens et al. 2018; De Leeuw et al. 2018), and are mostly suited to broad spatial and temporal scales (George et al. 2016; Troumbis e Iosifidis 2020). Conversely, citizen‐reported wildlife sightings to local authorities offer real‐time, fine‐scale data on where and when interactions occur (Quinn et al. 2016), along with information on public concerns. Although these reports tend to overrepresent negative perceptions and concern-eliciting encounters (Hayman et al. 2014; Wilbur et al. 2018), this bias makes them especially valuable for detecting patterns of public concern in contexts like wolf recolonization and for exploring how these patterns relate to both wolf- and human-related factors.

Indeed, the frequency of wolf reports likely reflects seasonal variation in wolf detectability, shaped by their ecology and affecting the likelihood of human encounters. In summer, wolf packs limit movements to denning areas with low human presence (Bassi et al. 2015), due to pups’ limited mobility (Ciucci et al. 1997; Roffler e Gregovich 2018), ultimately reducing detectability. In contrast, the mid-late winter peak of dispersal (Morales‐González et al. 2022), leading to broader movements (Fuller et al. 2003) and often lower human avoidance (Barry et al. 2020), may increase wolf detectability. This aligns with the winter peak in human-caused wolf mortality in Italy, mostly from accidental causes such as vehicle collisions (Musto et al. 2021), further supporting a seasonal increase in human–wolf interactions and, potentially, of the associated public concern.

Spatial variation in wolf detectability is also shaped by ecological factors beyond seasonal movements. Recently recolonized areas usually have lower wolf densities due to limited time for population growth (Pletscher et al. 1997; Mysłajek et al. 2018), while long-established areas often have higher densities of wolves (Mattioli et al. 2018). Moreover, although wolves persist in human-dominated landscapes, they typically prefer low-disturbance areas with abundant prey and cover (Ciucci et al. 1997; Bassi et al. 2015; Zanni et al. 2023). All else being equal, this heterogeneous wolf distribution (see also Figures S2, S3, S4, and S5 for wolf distribution within the study area of this study) should reduce report frequency and perceived concerns in urban and recently recolonized areas. However, human factors at the wolf–human interface can counteract patterns driven solely by wolf biology (Behr et al. 2017). In recently recolonized areas, we might expect higher wolf sighting reports due to the novelty of encounters. People tend to share events that strike them as unusual, as seen with urban wildlife reports during COVID-19 restrictions (Manenti et al. 2020; Zellmer et al. 2020; Murray et al. 2023). Conversely, in long-established areas, wolf sightings may no longer be seen as noteworthy. Higher human density in urban areas can also offset lower wolf density by increasing the number of observers, leading to more wolf reports through a density-dependent process, as shown in species distribution studies (Zizka et al. 2021). Importantly, this should not be considered a sampling bias if the goal is to understand where and when wolf–human interactions and related concerns arise, rather than to infer wolf distribution.

At the same time, human perceptions of wolf sightings may follow spatial patterns not necessarily aligned with the actual distribution of sightings. For instance, limited human–wolf conflicts in recently recolonized areas may reduce negative attitudes (Manfredo 2008, Ericsson et al. 2018, Barmoen et al. 2024, but see Zimmermann et al. 2001). At the same time, wolf presence in urban and suburban areas could challenge the traditionally more positive attitudes of urban citizens towards wolves (Ericsson et al. 2018; Pettersson et al. 2021; Barmoen et al. 2024), as these attitudes largely stem from the spatial separation typically maintained between wolves and urban citizens (Karlsson e Sjöström 2007). Moreover, as a symbols of wilderness in human cultures (Figari e Skogen 2011; Almarcha et al. 2022), wolves may amplify feelings of trespassing and intrusion when present in highly urbanized areas, heightening concerns and negative perceptions. This interplay of contrasting wolf- and human-related factors makes the upcoming scenarios of wolf–human interactions inherently unpredictable, underscoring the need for dedicated studies at finer spatiotemporal scales.

We located our study in Tuscany, central Italy, where wolves never completely disappeared (Cagnolaro et al. 1974), have long been monitored (Cagnolaro et al. 1974; Boitani e Ciucci 1996; Gazzola e Viviani 2006; Merli et al. 2023; Zanni et al. 2023), may reach locally high densities (Mattioli et al. 2018), and were recently reported near both small and large human settlements (Zanni et al. 2023). We analysed the time and location of occurrence and the valence (i.e. pleasure-displeasure responses, Barrett et al. 2007) of the wolf reports received by the Tuscany Region administration’s wolf report email, WhatsApp and phoneline service from 2021 to 2024. Employing an observed/availability design, we modelled the spatiotemporal distribution of wolf reports as a function of the time of year, recolonization history, and urbanization, measured via the human footprint (a comprehensive proxy for human presence and activities, Mu et al. 2022). Hypothesizing that these factors would influence both when and where wolf reports occur, we predicted:

(i) wolf reports to increase in late winter and decrease in summer (P1), following dispersal and reproductive seasonality;
(ii) wolf reports to be more frequent either in areas of long-term wolf presence (P2a), due to higher wolf density, or in recently recolonized areas (P2b), due to “wolf-novelty” effects;
(iii) wolf reports to be most frequent in areas with an intermediate human footprint (P3), where the spatial overlap between humans and wolves is likely greatest.

We further hypothesized that the spatial variation of human emotions towards of wolf sightings would be shaped by the recolonization history and the urbanization, predicting:

(i) a higher likelihood of negative valence reports in areas with long-term wolf presence (P4), due to a prolonged exposure to human–wolf conflicts;
(ii) a higher likelihood of negative valence reports in areas with higher human footprint (P5), as wolves in urban settings may challenge the traditionally positive attitudes of urban citizens and escalate feelings of trespassing and intrusion.

## Materials and methods

### Study area

**Fig. 1:**
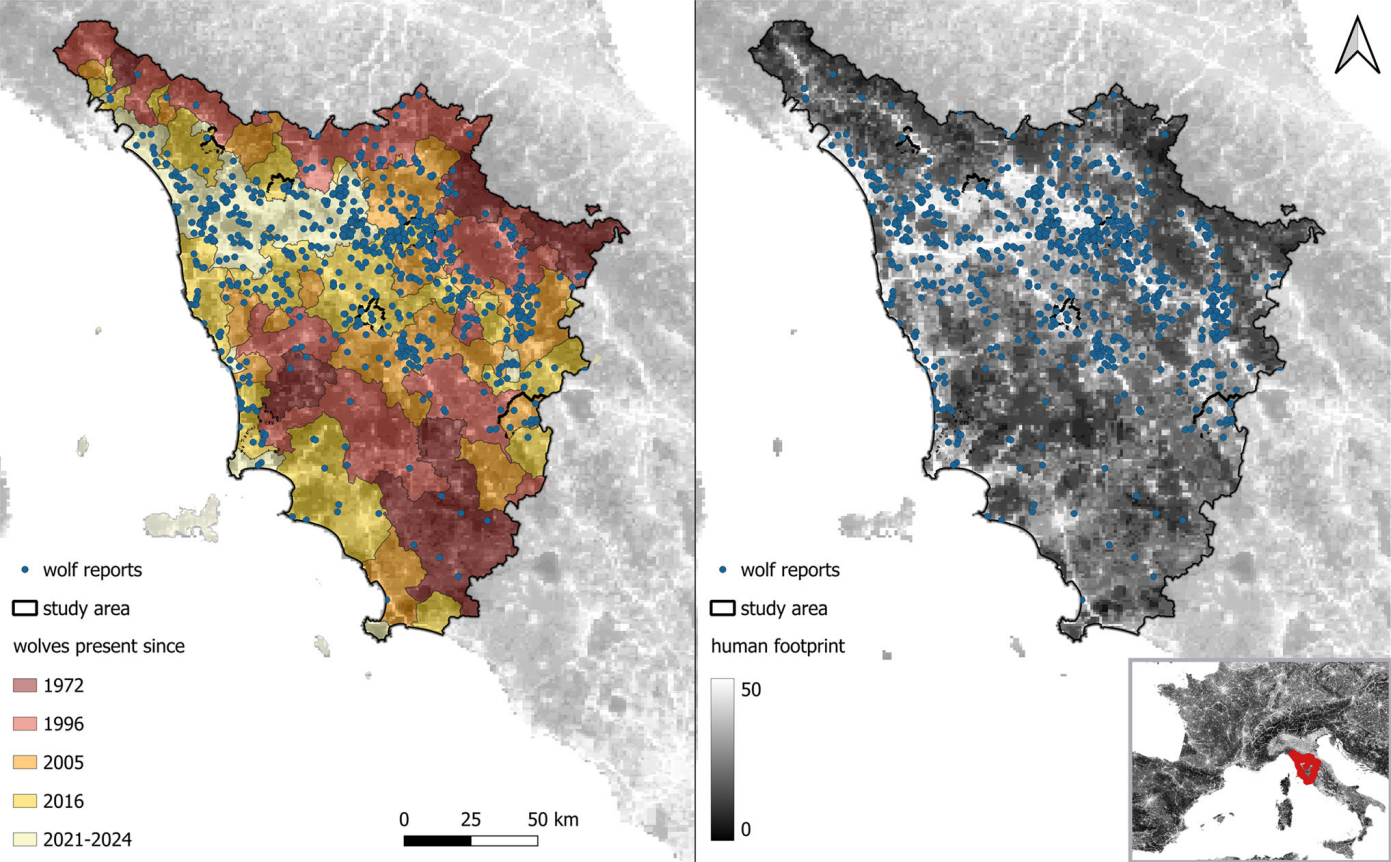
Wolf reports received by the Tuscany Region phoneline service in 2021–2024 (*n* = 914) and their spatial distribution across areas occupied by wolves in different years (left) and across the human footprint gradient (right). The bottom-right panel indicates the position of the study area (red) in southern Europe

The study was conducted in Tuscany (Fig. 1), a central Italian region with elevations ranging from sea level up to 2054 m above sea level. Forested areas, including shrublands and both broadleaved and coniferous forests, cover 52.7% of the region, while agricultural land accounts for 37.6%. Built-up areas, defined as surfaces occupied by artificial infrastructure such as residential and industrial buildings or paved roads, represent 4.9% of the landscape (Copernicus Land Monitoring Service 2018). The remaining surface consists of minor land-cover types such as water bodies and bare rock. Human presence and activity are widespread but spatially heterogeneous, with the human footprint averaging 25.44 but ranging from 2.5 to 50 across the region, nearly covering the full global scale of 0–50 (Mu et al. 2022).

Tuscany has a long history of continuous wolf presence and monitoring. The population reached its lowest historical levels in the 1970s, persisting in few localized areas (Cagnolaro et al. 1974). From the 1970s onward, wolves gradually recolonized additional territories (Boitani e Ciucci 1996; Gazzola e Viviani 2006), with confirmed reports of their presence in nearly 90% of the Region’s surface by 2016 (Merli et al. 2023; Zanni et al. 2023). The ranges reoccupied across successive monitoring occasions slightly tended to be characterized by progressively higher levels of human footprint (Figure S1). During the most recent survey conducted in 2016, which was primarily based on camera trapping and wolf howling techniques, wolf pack density (based on pack locations kindly shared upon request by Merli et al. 2023 and Zanni et al. 2023) was higher in areas of historic presence (Figures S2 and S4) and inversely related to the human footprint (Figures S3 and S5).

### Wolf report collection and classification

Due to the steady expansion of the wolf population of Tuscany, including around human settlements (Zanni et al. 2023), and the resulting rise in interactions and public concerns, in 2021 the Tuscany Region wildlife office launched a dedicated reporting service^1^ to monitor the spatiotemporal dynamics of wolf–human interactions and guide citizens in responding appropriately to wolf encounters. Contact details were shared through official regional media and disseminated to local law enforcement agencies and administrations, which helped redirect citizen reports to the appropriate contacts. Citizens could not report directly via the website but were encouraged to call a dedicated phone number or send a message via WhatsApp or email. The use of familiar communication channels increased accessibility across age groups and levels of digital literacy, although we acknowledge that some segments of the population may still have had limited access. Between July 2021 and July 2024, the service received 1079 reports. Reporters were informed about appropriate behaviour during wolf encounters and asked to provide the date and coordinates of their sighting, which were logged in a digital database. When exact dates were missing, we used the report submission date; if coordinates were absent, we georeferenced detailed location descriptions (e.g. toponyms, road intersections, or kilometre markers) using a regional cartographic tool.^2^ We considered all reports received: direct observations of wolves, signs of presence, suspected or confirmed wolf predation signs, and requests for action after a wolf sighting. We analysed only reports with ascertained coordinates received from July 12, 2021 (when the wolf reporting service became fully operational) to July 31, 2024, totalling 914 wolf reports.

Starting on January 1, 2022, we also qualitatively categorized the valence of reports (*n* = 705) based on the language used by the reporters, with the term valence used to denote the pleasure-displeasure spectrum of emotions towards wolves (Barrett et al. 2007). Reports were classified as negative (*n* = 323) if they either explicitly contained words associated with fear, anger, or concern (i.e. fear, anger, concern, danger, emergency, scare, or related terms), or implicitly conveyed these attitudes (e.g. statements like “we can’t even leave the house” or “there are children here”). Non-negative reports (*n* = 382) comprised those with a positive valence (e.g. expressing admiration for the sighting or concern for wolves’ safety) and neutral reports that simply provided information without emotional content (e.g. “I saw a wolf here yesterday”). Positive and neutral reports were merged into a single non-negative category due to the very low number of positive reports (*n* = 33). To ensure consistency in classification, we tested inter-observer reliability in coding the reports on 30% of the data, achieving complete agreement across two independent coders (GN, RB). This procedure ensured reproducibility and validity by confirming that coding decisions were not dependent on a single observer (Nili et al. 2020).

### Spatial covariates extraction

To study which geographical factors were associated both with the distribution of wolf reports and their valence, we categorized the entire study area in terms of (i) the time elapsed since wolf recolonization, and (ii) the human footprint (i.e. landscape urbanization).

As a measure of time since wolf recolonization and therefore the potential level of local people’s habituation to wolves, we employed a vector layer delineating municipalities where wolf presence was first confirmed in 1972, 1996, 2005, or 2016. This spatial dataset, combining a 2016 survey with earlier monitoring data from technical reports and publications, was kindly provided upon request by Zanni et al. (2023). We assigned a categorical variable (hereafter referred to as recolonization step) to each report location, corresponding to the year of wolf recolonization in that area. Reports in areas still uncolonized as of 2016 were assigned a fifth, most recent category.

We used a human footprint raster layer of 2020 (relative index ranging from 0 to 50, resolution of 1 km) provided by Mu et al. (2022) as a proxy of urbanization. The index employs varying weights for distinct categories such as urban structures, including residential, commercial, and industrial built-up areas, modified agricultural areas, transportation infrastructure including roads and railways, as well as nocturnal illumination. We assigned the local human footprint to all report locations. Finally, to account for the potential variance in the diffusion of the wolf report phone service across the ten provinces of Tuscany (administrative units, each including multiple municipalities), each report was assigned to the province in which it occurred.

### Modelling the spatiotemporal distribution of wolf reports

We modelled the spatiotemporal distribution of wolf reports by adopting an observed/availability design (Manly et al. 2002), comparing the characteristics of locations where wolves were actually reported (observed report locations) to a set of randomly selected locations that represent where reports could potentially occur (hereafter referred to as random report locations). By matching these observed and random locations in space and time, we were able to statistically evaluate how spatial factors influenced the likelihood of a wolf report being made across the landscape. Because the phone service was active across all of Tuscany, we generated random report locations throughout the entire region. For each observed wolf report, we sampled a set of random report locations within the Tuscany region, by means of the *spsample()* function of the *sp* R package and a spatial layer of the regional borders. Since possible wolf density effects were directly embedded in our prediction framework (P2a and P3), we did not constrain the generation of random report locations by wolf pack distributions, thereby keeping available points independent of underlying wolf densities. To account for seasonal variation of wolf reports stemming from the seasonality of wolf movements, we randomly assigned a date from 1 January to 31 December to each random report. Given that some reporters provided multiple wolf reports (with the 914 observed reports being provided by a total of 659 reporters), we assigned the identity of the reporter to each observed report, as well as to its corresponding random reports. To ensure stability in the model parameters, we defined the optimal number of random reports to be associated with each observed report by running a sensitivity analysis (Brogi et al. 2023), which suggested the use of 11 random reports per observed one. Subsequently, we introduced a new binary variable, “case”, to each location, with a value of 0 representing random reports and 1 representing observed reports.

We estimated the spatiotemporal variation of having wolf reports by running a binomial GLMM (generalized linear mixed model) with the *glmer()* function (*lme4* R package) and the optimizer *bobyqa*, with “case” as response variable and both the reporter identity and the province administration included as random effects. The input datasets comprised the whole set of observed (914) and random (10 054) report locations, for a total of 10 968 entries. In line with our hypotheses, we aimed at testing the effects of three main factors on the spatiotemporal distribution of wolf reports: the seasonality, the recolonization step, and the local human footprint. We modelled the seasonal distribution of wolf reports using the approach by Stolwijk et al. (1999) to account for the continuity between 31 December and 1 January. Dates were converted to Julian days, scaled to radians (Julian day/365 × 2π), and then included in the model as sine and cosine terms. The recolonization step, representing progressive time steps, was modelled as an ordinal categorical variable with five levels (1–5), reflecting the inherent temporal ordering of the steps. This approach allowed the model to capture nonlinear effects on the response, without assuming equal spacing between recolonization steps, with polynomial contrasts being applied to account for potential higher-order effects. Finally, since we hypothesized that wolf reports may have been most frequent at intermediate levels of human footprint, we included both the linear and squared terms of human footprint to capture potential nonlinear effects. Since we had repeated reports from the same reporter and several within the same province, we included the reporter identity and the provincial administration as random intercept effects. To keep type I error rate at the nominal level of 0.05, we included random slopes (Schielzeth e Forstmeier 2009; Barr et al. 2013) of all the predictors within both reporter identity and provincial administration into the model. Prior to fitting the model, we screened all predictors for collinearity (Pearson coefficient |r_p_|< 0.7) and multicollinearity (Variance Inflation Factor, VIF < 3, Zuur et al. 2009), but no issues arose (r_recoloniszation step, human footprint_ = 0.44). After this, we z-transformed the human footprint to a mean of zero and a standard deviation of one to get comparable estimates and easier interpretable model results (Schielzeth 2010). With a dispersion parameter of 0.883 the response was not overdispersed.

As an overall test on the effect of seasonality, recolonization step, and human footprint on the spatiotemporal distribution of wolf reports and to avoid “cryptic multiple testing” (Forstmeier e Schielzeth 2011), we compared the fit of the full model as described above with that of a null model lacking the five fixed effects but being otherwise identical, by means of a Chi-squared test (*anova()* R function). If the full model showed a significant effect, we proceeded to test the significance of each of the three factors individually through comparisons with reduced models, each lacking the term(s) associated with a specific factor (Barr et al. 2013). Specifically, we tested seasonality by removing the sine and cosine terms of the Julian date expressed in radians, recolonization step by removing the polynomial contrasts for this factor, and human footprint by removing its linear and quadratic terms simultaneously. Finally, we tested the nonlinear effect of the human footprint by comparing the full model with a reduced model lacking its square term but including its main effect.

Models estimated with observed/available data pose unique challenges when assessing model predictions, as these are not true presence/absence data. We thus used a fivefold cross-validation method tailored for observed/available designs (Boyce et al. 2002), calculating a Spearman rank correlation between model ranks and area-adjusted frequencies in withheld data subsets (1/5 of the dataset). Strong predictive models would show high positive correlations (Wiens et al. 2008).

### Modelling the valence of wolf reports

To quantify how human footprint and recolonization history affected the valence of each wolf report, we used a generalized additive model (Zuur 2012) with a binomial distribution, to predict the probability that each report had a negative valence.

We used the human footprint and recolonization step as covariates in our GAM. The first was z-transformed to a mean of zero and a standard deviation of one to get comparable estimates and easier interpretable model results (Schielzeth 2010), while the second was treated as an ordinal covariate with orthogonal contrasts, in line with the protocol used for the spatiotemporal distribution analysis. Considering that 47.3% of wolf reports were unique contributions, with each submitted by a single individual, it was not possible to fit a multilevel model with a random intercept and/or slope structure to account for inter-individual variability in the valences of each call. Similarly, we did not include province as a random effect, as we had no theoretical expectation of spatial variation in report valence across administrative areas.

We used a stepwise model selection approach, adding each covariate sequentially and testing its significance with likelihood-ratio tests and visual inspection of effect sizes (Cumming 2014). For the human footprint, we compared spline types and basis dimensions using Akaike’s Information Criterion (AIC), selecting an adaptive spline with *k* = 25. Moreover, we controlled for residual spatial correlation through a bivariate thin-plate spline with *k* = 40 that modelled the effect of the coordinates of each wolf report over the probability that reports had a negative valence. Finally, to obtain an overall measure of accuracy for our best candidate model, we calculated the area under the curve (AUC). This approach was repeated 1000 times after having divided our data randomly into a train (70%) and test (30%) datasets every time. Model fitting was based on a restricted maximum likelihood estimation, as this approach is considered suitable for relatively low sample size (*n* < 1000, Wood 2017).

## Results

The number of wolf reports received steadily increased over the study period, with an average daily rate of 0.28 reports in 2021, rising to 0.61 in 2022, 1.07 in 2023, and 1.21 in 2024 (Figure S6). The 914 wolf reports were unevenly distributed across the five recolonization steps, with densities of 0.92, 1.92, 5.38, and 4.39 reports per 100 km^2^ in areas where wolf presence was established as early as 1972, 1996, 2005, and 2016, respectively. The density peaked at 9.47 reports per 100 km^2^ in areas where wolf presence is newly documented by this study, highlighting that wolves have now recolonized the entire region of Tuscany, excluding islands, as also illustrated in Fig. 1. While 49.50% of wolf reports were neutral, it was possible to assign a negative or positive valence to 50.50% of reports: 90.73% of these had clearly negative valence, while the remaining 9.27% conveyed positive emotions or perceptions of the sightings.

### Spatiotemporal distribution of wolf reports

**Fig. 2:**
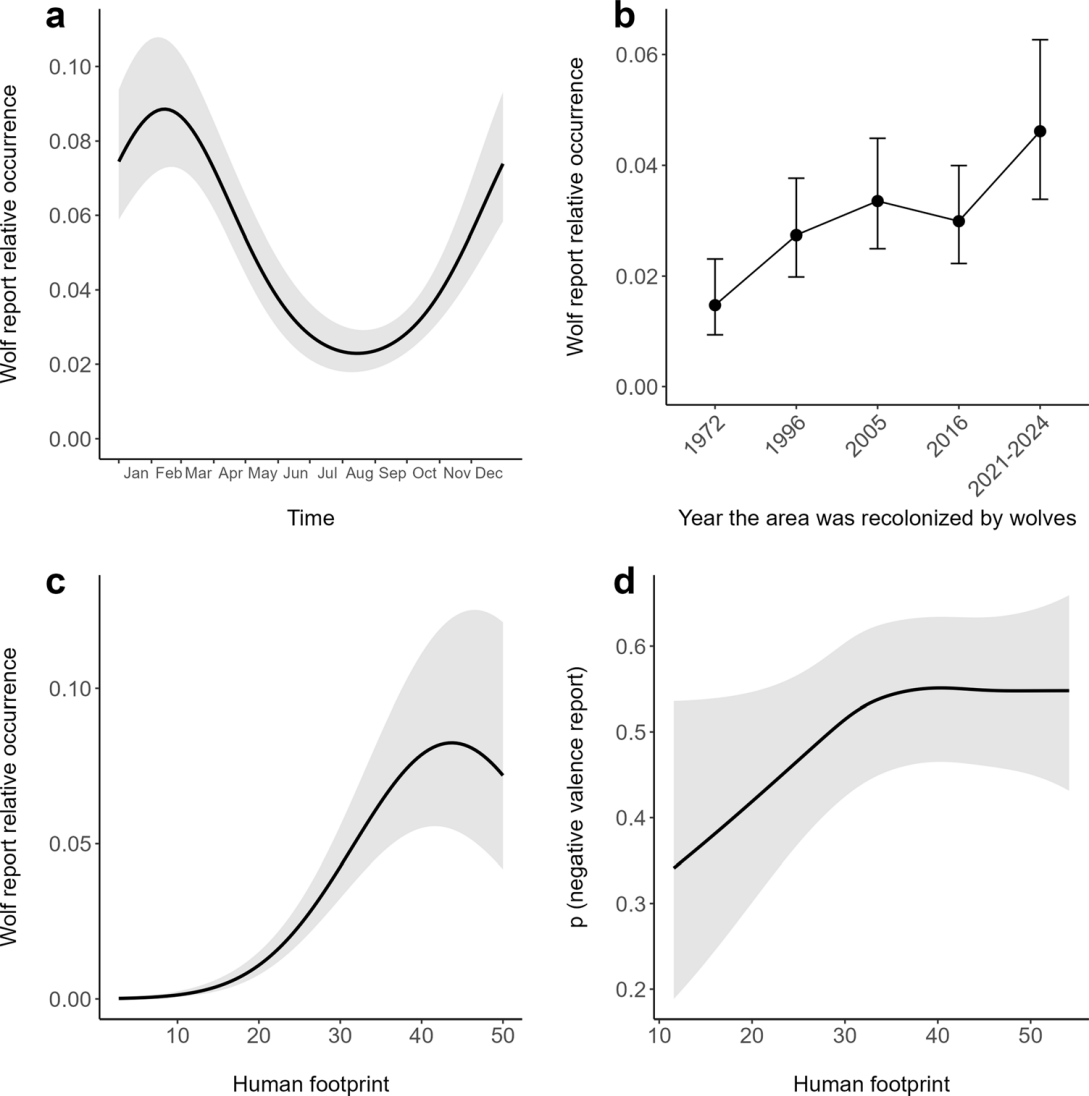
Variability in wolf report patterns as predicted by statistical models: **a** occurrence of received reports along the year, *P* < 0.001, **b** occurrence of received reports across areas recolonized in different years, *P* < 0.001, **c** occurrence of received reports along the human footprint gradient, *P* < 0.001 (panels a–c: Generalized Linear Mixed Model; see “Spatiotemporal distribution of wolf reports” section for details), and **d** likelihood of a wolf report revealing a negative valence along the human footprint gradient, *P* < 0.001 (Generalized Additive Model; see “Valence of wolf reports” section for details). Grey-shaded areas or vertical bars represent 95% confidence intervals

Overall, the full model including the Julian day, the recolonization step and the human footprint was clearly significant as compared to the null model (likelihood ratio test: χ2 = 222.42, df = 8, *P* < 0.001) and had outstanding predictive ability on withheld data (Spearman correlation coefficients: ρ_fold1_ = 0.976, ρ_fold2_ = 0.964, ρ_fold3_ = 0.976, ρ_fold4_ = 0.964, ρ_fold5_ = 0.961), revealing clear spatiotemporal variations of wolf reports accountable to the seasonality, the recolonization step, and/or the local human footprint. The other comparisons revealed that each considered factor had a significant overall effect (likelihood ratio tests comparing full and reduced models: χ^2^ = 69.781, df = 2, *P* < 0.001 for seasonality; χ^2^ = 38.124, df = 4, *P* < 0.001 for recolonization step; and χ^2^ = 103.708, df = 2, *P* < 0.001 for human footprint). Specifically, wolf report occurrence varied significantly throughout the year, with the lowest values observed between August and September, followed by a notable threefold increase between February and March (Fig. 2a). The model also predicted an increasing trend in wolf report occurrence across recolonization steps, with the highest relative occurrence recorded in regions recolonized after 2016 (Fig. 2b). Finally, wolf report occurrence increased up to human footprints of 43.80 and then stabilized (Fig. 2c), with a highly significant nonlinear effect (χ^2^ = 82.086, df = 1, *P* < 0.001).

### Valence of wolf reports

Our best candidate model to predict the probability that a certain wolf report had a negative valence included the human footprint and the recolonization step as predictors (AUC = 0.6 ± 0.03). The local human footprint had a significant effect (χ^2^ = 40.488, df = 14.59, *P* < 0.001), with the probability of a certain record having a negative valence increasing until intermediate values of it (38.01), and then reaching a plateau (Fig. 2d). Although the likelihood ratio test did not identify a significant effect of the wolf recolonization step (χ^2^ = 8.820, df = -3.51, *P* = 0.06), the analysis of conditional effects indicates that the probability that reports had a negative valence tended to be lower in areas that were colonized more recently by wolves (Figure S7).

## Discussion

Our study highlights the potential value of citizen reports for mapping spatiotemporal patterns of wolf–human interactions and associated concerns across landscapes that differ both in the timing of wolf recolonization and in levels of human footprint. The collected reports revealed a novel landscape of wolf-related concerns, shaped by the interplay of wolf- and human factors. The seasonality of wolf dispersal and reproductive behaviours influenced temporal patterns of report frequency. Spatial variability, on the other hand, was consistent with expectations based on human factors, such as higher report rates in areas of recent recolonization and elevated human footprint, but contrasted with patterns that would be expected based on the likely wolf distribution.

Our results show a pronounced late-winter peak in wolf reports, which is consistent with P1 and with the documented seasonal patterns of wolf movements (Roffler e Gregovich 2018; Morales‐González et al. 2022) and the seasonality of human-driven mortality of wolves (Musto et al. 2021). Since these represent the drivers and consequences, respectively, of wolf–human interactions, their alignment with the observed seasonality of wolf reports supports the reliability of citizen reports as a proxy for mapping wolf–human interactions. The higher frequency of winter reports likely reflects increased wolf movements after pup-rearing (Roffler e Gregovich 2018), the wider-ranging movements of dispersing wolves (Fuller et al. 2003), and their reduced human avoidance (i.e. a weaker tendency to avoid roads and human settlements during dispersal, Barry et al. 2020), highlighting wolf behaviour as a key driver of seasonal patterns of human–wolf interaction and potentially allowing us to predict a winter peak in wolf-related concerns.

The spatial distribution of wolf sighting reports did not follow the locally expected wolf density, revealing a major role played by human factors, including the duration of coexistence with wolves and the local human footprint. Areas recolonized more recently by wolves hosted a higher frequency of wolf reports, consistent with our prediction about the wolf-novelty effect (P2b). This pattern suggests that the novelty of wolf presence in these areas increases human awareness and reporting behaviour, resulting in a higher frequency of sighting reports despite the likely lower wolf densities (Figure S4). Similarly, most reports originated from areas with moderate-to-high human footprint, with frequencies rising until they plateaued in the most urbanized settings. This pattern partially supports our prediction that wolf reports would be most frequent in areas with an intermediate human footprint due to greater spatial overlap between humans and wolves (P3), although the observed peak shifted towards medium–high levels of human footprint. The higher occurrence of reports in these areas may simply reflect their relatively large human population and, consequently, a large pool of potential reporters, but also reveals a notable wolf presence, at least up to medium–high levels of human footprint. In this context, the final descending trend in reporting may actually reflect the limited presence of wolves in the innermost parts of urban centres (Figure S5), limiting both interactions and reporting opportunities. However, wolves may have also been more easily detected in urbanized contexts, as they are drawn to human settlements to scavenge on waste (Mohammadi et al. 2019) or to prey on dogs or pets in general. Highly urbanized areas might even host individuals less fearful of humans (Newsome et al. 2017) and thus more easily detectable, possibly due to behavioural plasticity or adaptations, as observed in other canids like coyotes (Breck et al. 2019) and red foxes (*Vulpes vulpes*, Morton et al. 2023, Lazzaroni et al. 2024). If individuals with greater tolerance for humans are more likely to persist in urban areas, this could further increase the likelihood of human encounters and related concerns. Further research on behavioural changes in urban wolves is therefore essential, given their potential implications not only for population and recolonization dynamics, but also for shaping public attitudes towards the species. Regardless of the underlying drivers, results indicate that most wolf–human interactions perceived as concerning by citizens occur, and can therefore be expected, in newly wolf-colonized and more urbanized areas, rather than where wolf densities are highest.

Report valence revealed predominantly negative perceptions of wolf sightings, highlighting a link between their spatiotemporal distribution and public concerns. However, these concerns were unevenly distributed across the collected reports, with sightings in areas with a higher human footprint more frequently eliciting reports with a negative valence. While this might seem inconsistent with the generally positive attitudes towards wolves often reported among urban residents (Ericsson et al. 2018; Pettersson et al. 2021; Barmoen et al. 2024), it aligns with the notion that such attitudes are largely contingent on spatial separation from wolves (Karlsson e Sjöström 2007). The observed patterns may also partially stem from the fact that we measured reactions to tangible wolf presence rather than abstract attitudes towards the species. Additionally, sightings in urbanized areas could have amplified concerns due to the stark contrast between these environments and the cultural symbolism of wolves as emblems of wilderness (Figari e Skogen 2011; Almarcha et al. 2022). Consistent with P5, the presence of wolves in urban and suburban areas, bringing urban residents into direct contact with the associated challenges of wolf–human coexistence, may disrupt the traditional divide in public opinions on wolf management and conservation between rural and urban populations. This result also depicts a novel scenario in which public concerns about wolves are no longer confined to traditional activities such as livestock breeding and hunting, predominantly associated with rural areas, but are increasingly shifting to common citizens residing in urban areas. Conversely, the limited sighting reports from areas occupied the longest exhibited the highest levels of fear, anger, and/or concern towards wolves, while areas with mid-term recolonization and newly colonized areas displayed a relatively flat trend in negative perceptions. Although the effect was too weak to support that long-term wolf presence exacerbates concerns (P4), the results highlight that long-lasting wolf presence did not increase tolerance in human populations. This finding contrasts with Zimmermann et al. (2001) but aligns with Ericsson et al. (2018) and Barmoen et al. (2024), indicating that long-standing exposure to wolves does not necessarily alleviate concerns.

While our study provides valuable insights into the spatiotemporal dynamics of wolf reports and human perceptions, it also entails limitations. First, citizen-reported data may introduce biases, such as the potential uneven willingness to report wolf interactions across individuals with different experiences and demographic characteristics, as seen in other potentially dangerous species (alligator, *Alligator mississippiensis*, Hayman et al. 2014; black bear, *Ursus americanus*, Wilbur et al. 2018). Although this could underrepresent less-concerned reports, our approach still effectively mapped wolf–human interactions that were perceived as concerning by citizens, making it a reliable tool for predicting the distribution of wolf-related concerns. Second, we recognize that the binary classification of report valence oversimplified the complex spectrum of human emotions and attitudes towards wolves. This limitation, however, arises from the nature of the data, which was gathered spontaneously in real-time to map interactions and resulting concerns, rather than to capture the full range of public attitudes towards wildlife, which falls beyond the scope of this study. These factors may contribute to an emphasis on concern-driven reports and a relative oversimplification of more neutral or positive reactions to wolf presence. Nevertheless, given the role of public concern in shaping political decision-making and support for wolf conservation (Birkland 1998; Treves et al. 2013; Slagle et al. 2022; Capitain et al. 2025), the distribution of negative reports may signal reduced acceptance of implemented management strategies, weakened political backing for wolf conservation, and even a growing potential for more conflictual human responses to wolf presence, particularly in urban areas, where such concern has traditionally been lower.

## Conclusions

Overall, our results illustrate a higher occurrence of wolf–human interactions during winter months and in areas of recent wolf expansion, with citizens in the most urbanized regions reporting more frequent sightings accompanied by heightened concern. Emerging from the interplay between wolf- and human-related factors, this multifaceted landscape of interactions could not have been fully understood through wolf biology and habitat suitability alone. By integrating both components, our approach effectively identified hotspots of wolf–human interactions in the most urbanized regions, thereby guiding the prioritisation of targeted socio-ecological management plans in these human-dominated landscapes. Being based on one of the European regions where wolf recovery began earliest and achieved the greatest success (Mattioli et al. 2018; La Morgia et al. 2022; Merli et al. 2023; Zanni et al. 2023), our results may foreshadow similar dynamics in areas of more recent, and ongoing, recovery. The observed shift in public perception, with growing concerns about wolves even among traditionally protectionist urban citizens, signals a crucial inflection point that may lead to declining public support and increased resistance to wolf conservation measures.

## Supplementary Information

Below is the link to the electronic supplementary material.

Supplementary file1 (PDF 1247 kb)

## Data Availability

Research data (i.e., geographical coordinates) could not be deposited in public repositories due to privacy concerns, as sightings were often reported from personal addresses and residences.

## Footnotes

1 https://www.regione.toscana.it/-/conoscere-il-lupo.

2 https://www.regione.toscana.it/-/geoscopio.
