# Supplementary material for: Wolves on the phone: Public calls reveal a rise in urban concerns as wolves recolonize human-dominated areas
Source: Ambio. 2025 Oct 18;55(4):936–47. doi: 10.1007/s13280-025-02264-z (PMC12961086; doi:10.1007/s13280-025-02264-z)
Supplement: Supplementary file 1 — Supplementary file1 (PDF 1247 kb) [file 13280_2025_2264_MOESM1_ESM.pdf]

Supplementary Information: this Supplementary Information has not been peer reviewed

Title: Wolves on the phone: public calls reveal a rise in urban concerns as wolves recolonize human-dominated areas

AMBIO, A JOURNAL OF ENVIRONMENT AND SOCIETY 2025

Wolves on the phone: public calls reveal a rise in urban concerns as wolves recolonize human-dominated areas

Supplementary Information

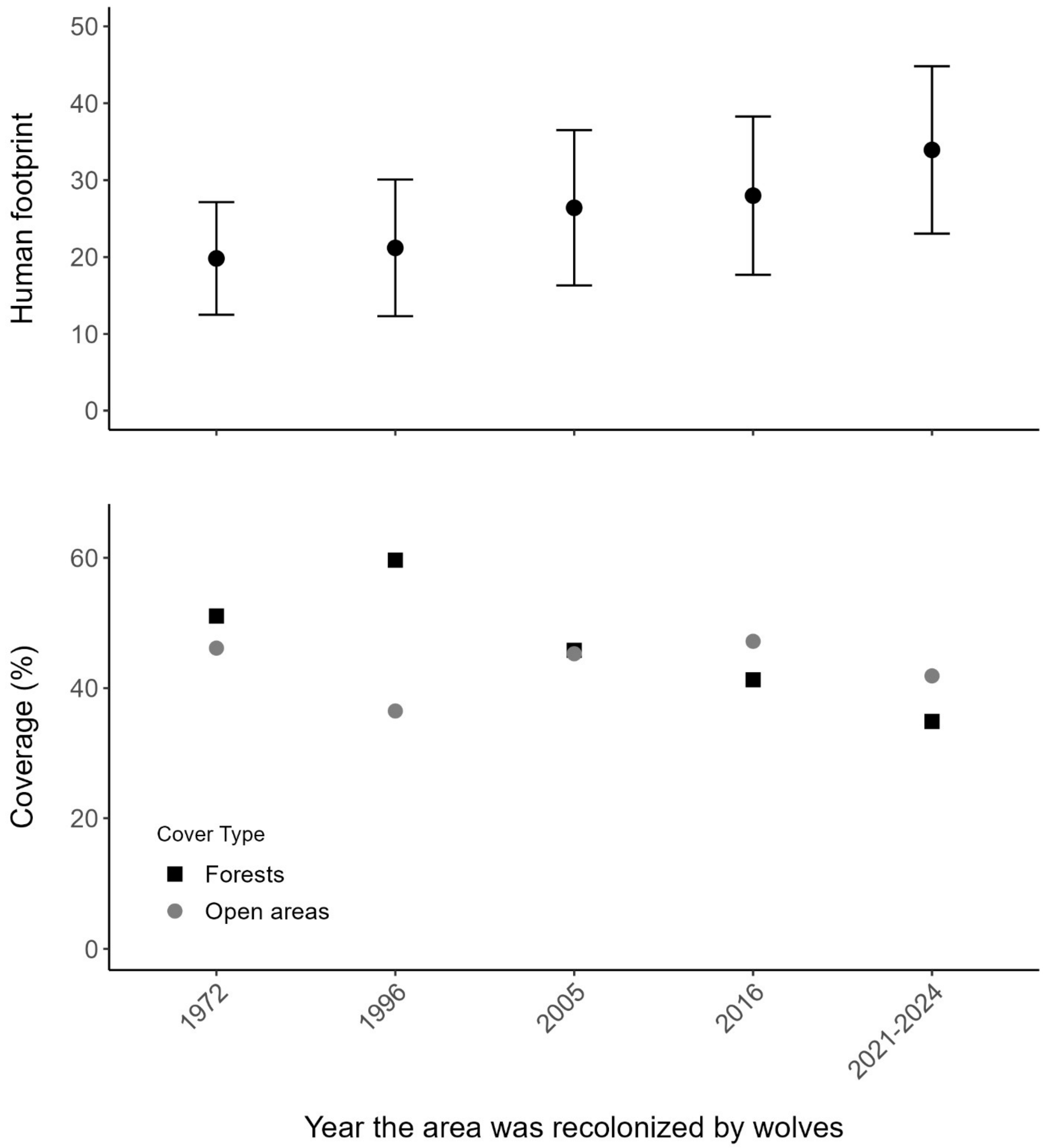

Figure S1. Landscape characteristics across wolf recolonisation phases in Tuscany, Italy. Top: mean human footprint (scale 0–50, Mu et al. 2022) for areas recolonised in different years, with vertical bars indicating one standard deviation. Bottom: percentage cover of forest and open land derived from the CORINE Land Cover database. Forest cover includes all highly vegetated classes (shrubland, deciduous, coniferous and mixed forests), while open land comprises natural and agricultural classes with sparse vegetation (pasture, arable land and bare rock).

Wolves on the phone: public calls reveal a rise in urban concerns as wolves recolonize human-dominated areas

Supplementary Information

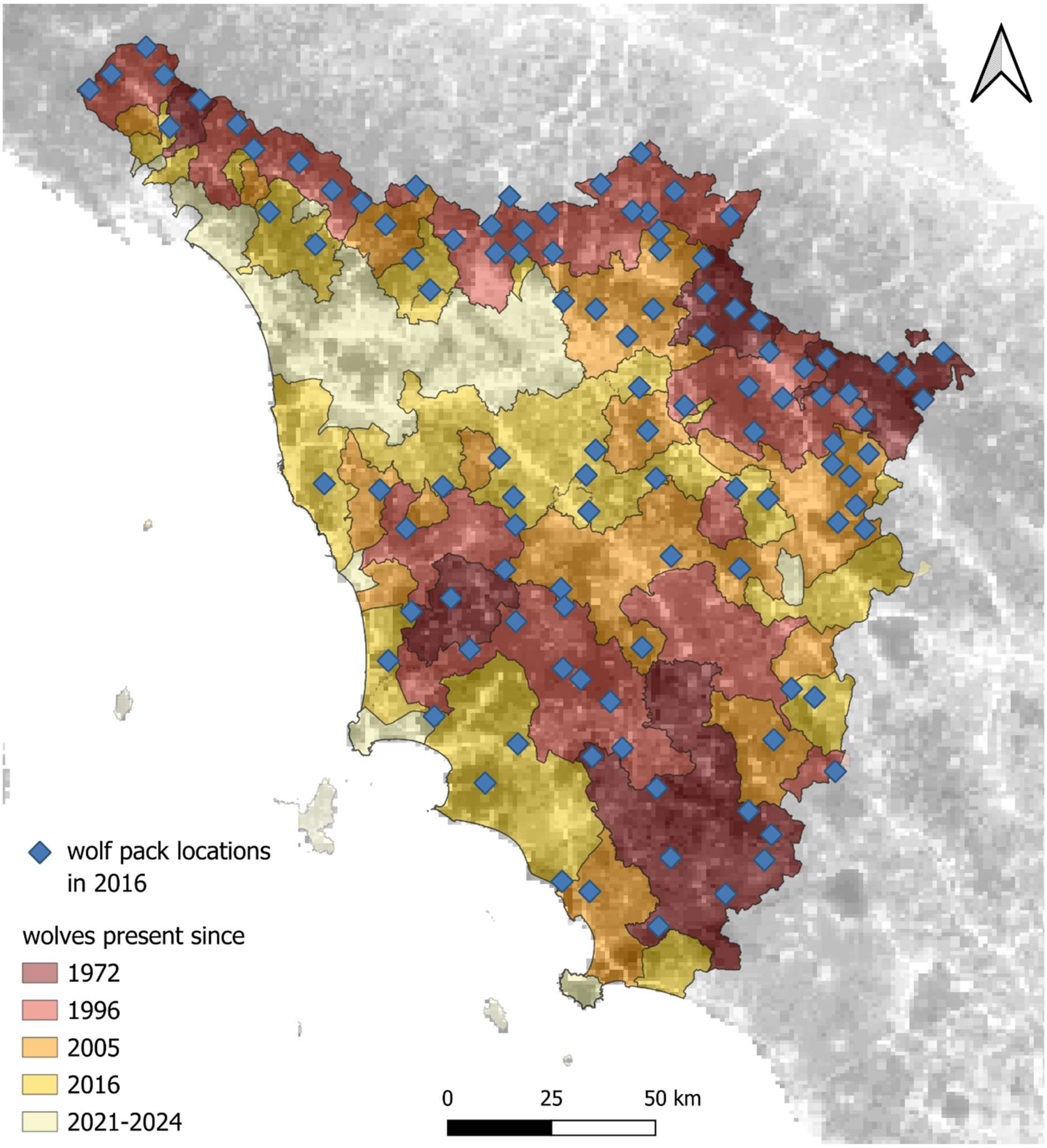

Figure S2. Wolf pack (blue diamonds, n=110) distribution in Tuscany, Italy, based on the latest regional survey conducted in 2016 (data provided by Zanni et al. 2023 and Merli et al. 2023 upon request). Packs were defined as social units including at least a territorial pair. Locations correspond to known rendezvous sites or, when unknown, to the site with the highest number of adult detections (e.g. the most frequently used camera trap, see Zanni et al. 2023 for details). Background colours indicate the year of wolf recolonization at the municipality level.

Wolves on the phone: public calls reveal a rise in urban concerns as wolves recolonize human-dominated areas

Supplementary Information

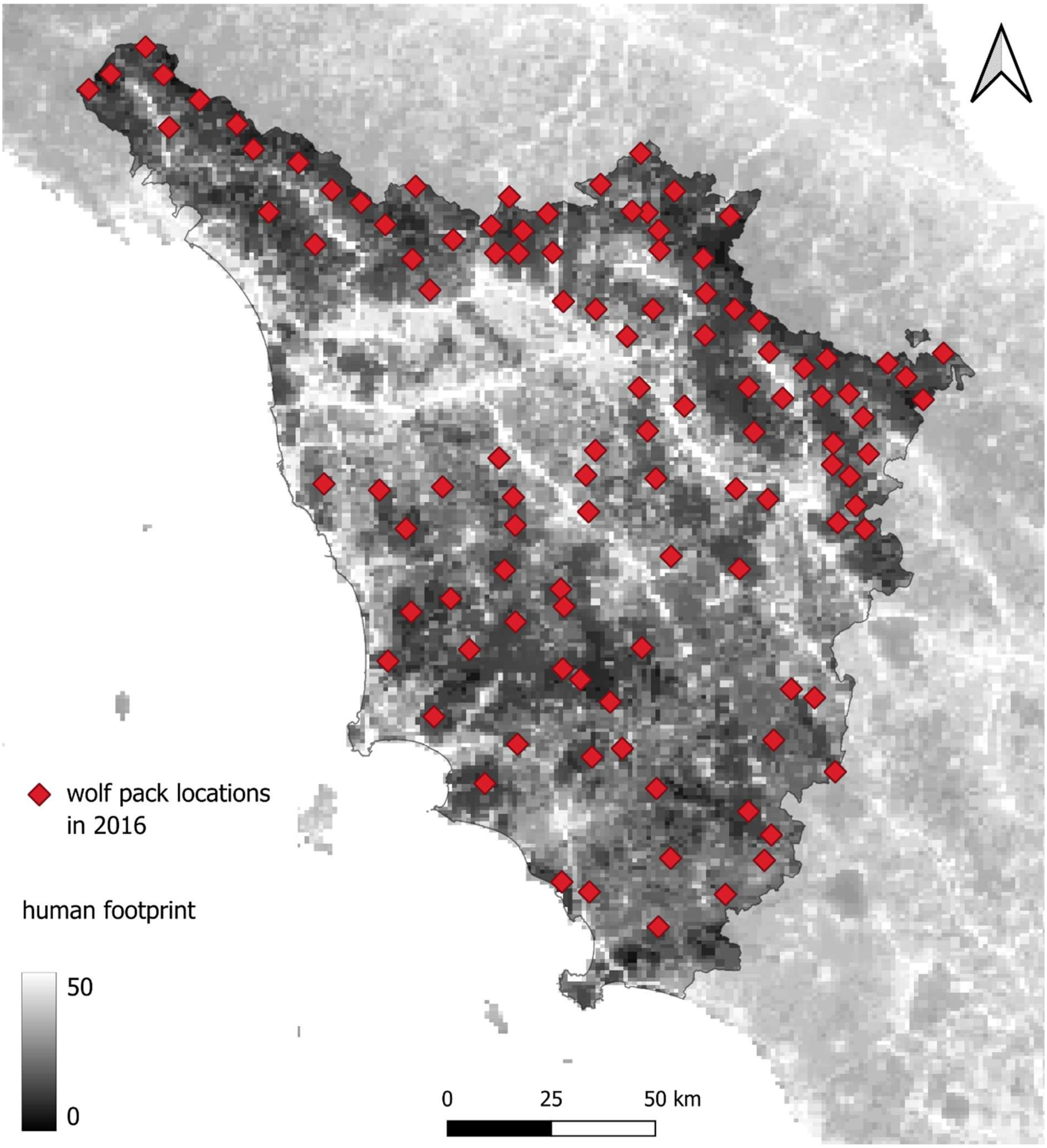

Figure S3. Wolf pack (red diamonds, n=110) distribution in Tuscany, Italy, based on the latest regional survey conducted in 2016 (data provided by Zanni et al. 2023 and Merli et al. 2023 upon request). Packs were defined as social units including at least a territorial pair. Locations correspond to known rendezvous sites or, when unknown, to the site with the highest number of adult detections (e.g. the most frequently used camera trap, see Zanni et al. 2023 for details). The background colour represents the human footprint (Mu et al. 2022).

Wolves on the phone: public calls reveal a rise in urban concerns as wolves recolonize human-dominated areas

Supplementary Information

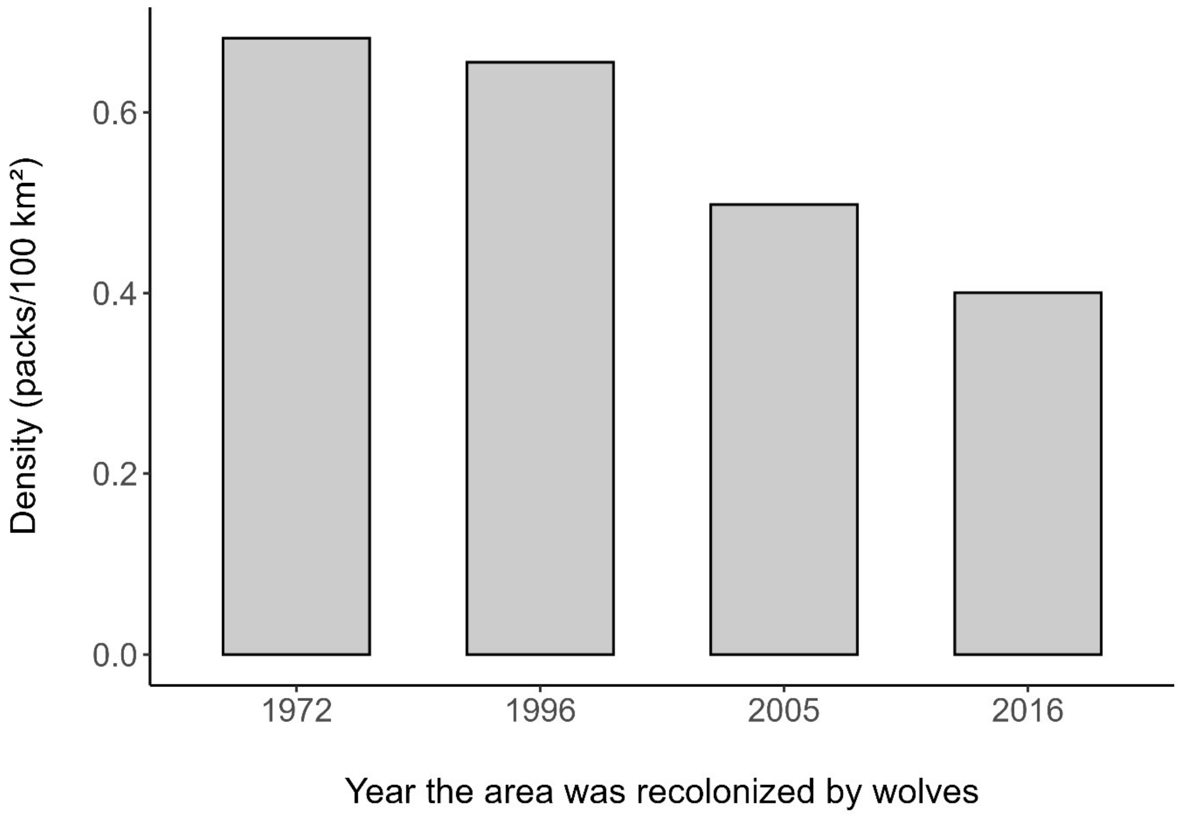

Figure S4. Wolf pack density among the recolonization steps in Tuscany, Italy, based on the latest regional survey conducted in 2016 (data provided by Zanni et al. 2023 and Merli et al. 2023 upon request). Pack density is expressed as the number of recorded packs relative to the total regional area within each recolonization step range (i.e., areas recolonized in a given year).

Wolves on the phone: public calls reveal a rise in urban concerns as wolves recolonize human-dominated areas

Supplementary Information

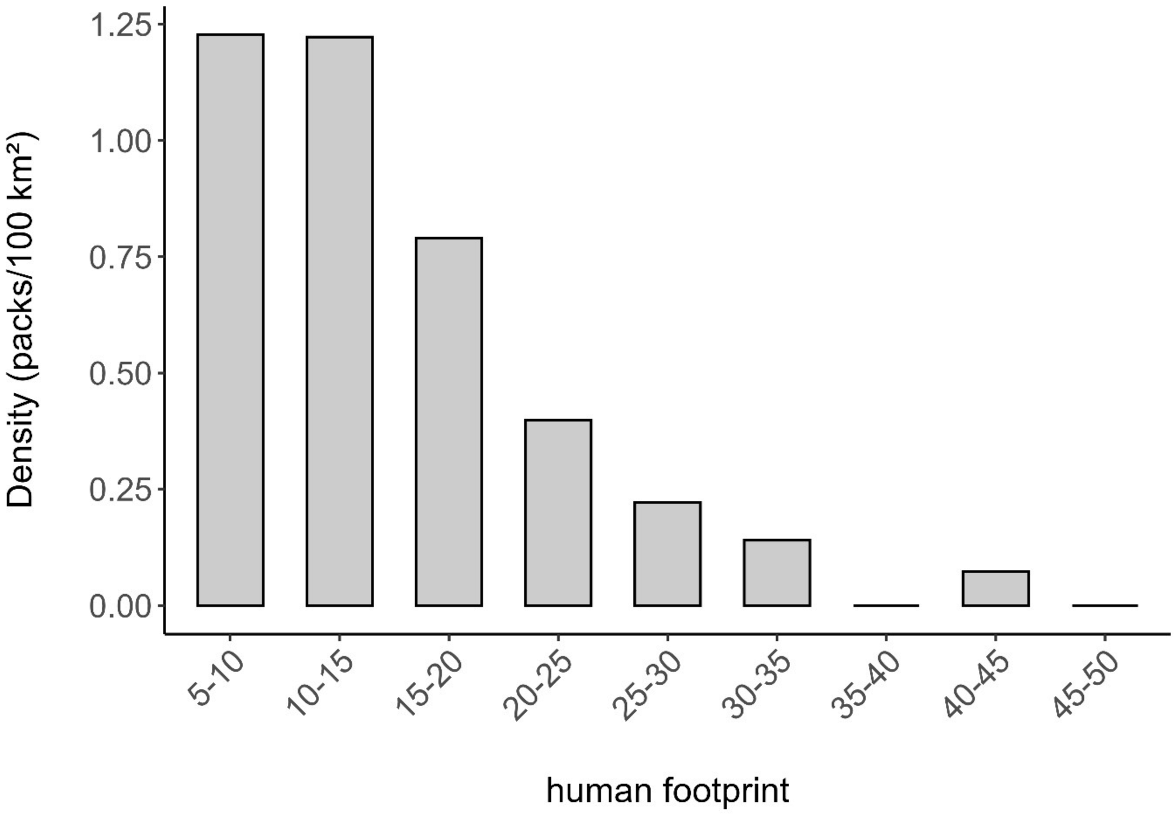

Figure S5. Wolf pack density along the human footprint (Mu et al. 2022) in Tuscany, Italy, based on the latest regional survey conducted in 2016 (data provided by Zanni et al. 2023 and Merli et al. 2023 upon request). Pack density is shown as the number of recorded packs per total regional area within each human footprint interval. Human footprint values were grouped into 5-unit intervals, right-closed (e.g., a value of 10 falls within the 5–10 range). The 0–5 interval was excluded from the visualization due to its minimal representation in the region.

Wolves on the phone: public calls reveal a rise in urban concerns as wolves recolonize human-dominated areas

Supplementary Information

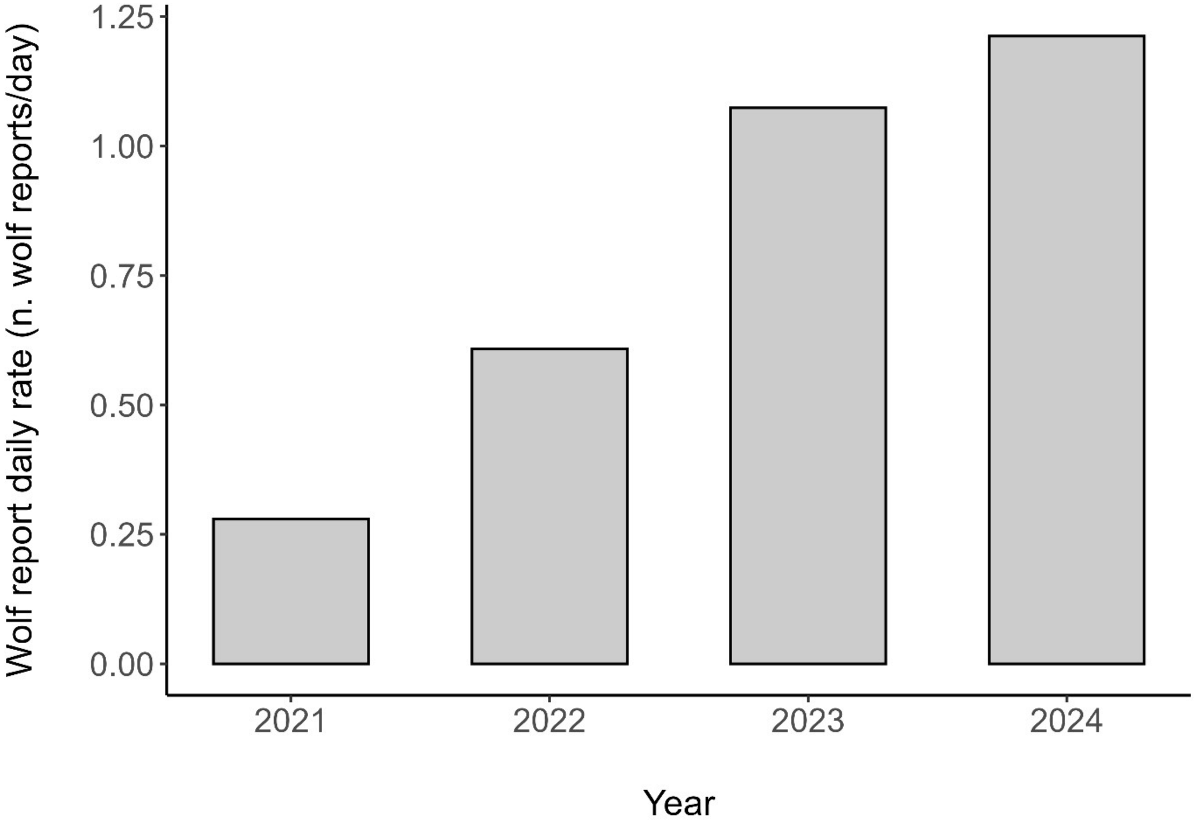

Figure S6. Daily rate of wolf reports received by the Tuscany Region reporting platform during the years of the study (see the main text for details).

Wolves on the phone: public calls reveal a rise in urban concerns as wolves recolonize human-dominated areas

Supplementary Information

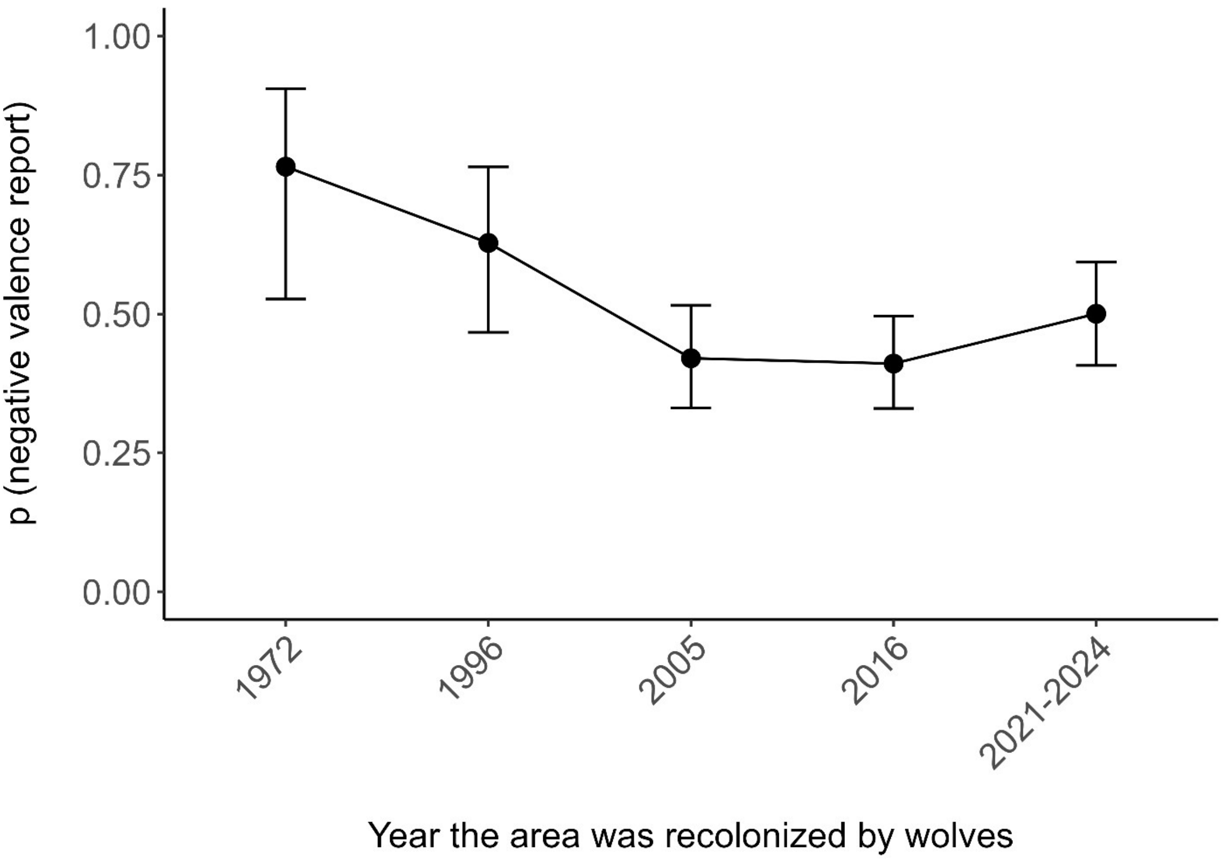

Figure S7. Variability of the likelihood of a wolf report revealing a negative valence across areas recolonized in different years (P=0.06) as predicted by the Generalized Additive Model (see section 3.2 of the main text for more details). Vertical bars represent the 95% confidence intervals.
